# Identification of Two Flavonoids as New and Safe Inhibitors of Kynurenine Aminotransferase II via Computational and In Vitro Study

**DOI:** 10.3390/ph18010076

**Published:** 2025-01-10

**Authors:** Redouane Rebai, Luc Jasmin, Abdennacer Boudah

**Affiliations:** 1 Department of Natural and Life Sciences, Faculty of Exact Sciences and Natural and Life Sciences, University Mohamed Khider of Biskra, BP145 RP, Biskra 07000, Algeria; 2 Laboratory of Biotechnology, National Higher School of Biotechnology, Ville Universitaire (University of Constantine 3), Ali Mendjeli, BP E66, Constantine 25100, Algeria; 3 Department of Oral and Maxillofacial Surgery, University of California, San Francisco, 707 Parnassus Ave Suite D-1201, San Francisco, CA 94143, USA; luc.jasmin@ucsf.edu

**Keywords:** herbacetin, (-)-Epicatechin, KAT-II inhibition, molecular docking, molecular dynamics

## Abstract

**Background/Objectives:** Kynurenine aminotransferase II (KAT-II) is a target for treating several diseases characterized by an excess of kynurenic acid (KYNA). Although KAT-II inactivators are available, they often lead to adverse side effects due to their irreversible inhibition mechanism. This study aimed to identify potent and safe inhibitors of KAT-II using computational and in vitro approaches. **Methods:** Virtual screening, MM/GBSA, and molecular dynamics simulations were conducted to identify the top drug candidates, followed by kinetic measurements and in vitro cytotoxicity evaluation. **Results:** The study showed that two compounds, herbacetin and (-)-Epicatechin exhibited the best scores. Their Glide docking scores are −8.66 kcal/mol and −8.16 kcal/mol, respectively, and their MM/GBSA binding energies are −50.30 kcal/mol and −51.35 kcal/mol, respectively. These scores are superior to those of the standard inhibitor, PF-04859989, which has docking scores of −7.12 kcal/mol and binding energy of −38.41 kcal/mol. ADMET analysis revealed that the selected compounds have favorable pharmacokinetic parameters, moderate bioavailability, and a safe toxicity profile, which supports their potential use. Further, the kinetic study showed that herbacetin and (-)-Epicatechin are reversible KAT-II inhibitors and exhibit a competitive inhibition mechanism. Their half-maximal inhibitory concentrations (IC50) are 5.98 ± 0.18 µM and 8.76 ± 0.76 µM, respectively. The MTT assay for cell toxicity indicated that the two compounds do not affect HepG2 cell viability at the necessary concentration for KAT-II inhibition. **Conclusions:** These results suggest that herbacetin and (-)-Epicatechin are suitable for KAT-II inhibition and are promising candidates for further development of KAT-II inhibitors.

## 1. Introduction

Tryptophan (TRP), one of the nine essential amino acids, is metabolized into several bioactive molecules. The most well-known is serotonin, produced from TRP in a separate pathway. However, up to 95% of TRP is transformed into kynurenine (KYN) and its breakdown products, including KYNA, via the KAT isozymes. An increase in kynurenic acid (KYNA) concentration in the brain is linked to schizophrenia and other CNS disorders [1,2]. Therefore, compounds that target the kynurenic pathway (KP) are of interest in developing drugs for CNS disorders.

In the human brain, KYNA is produced through KAT-II, making this enzyme an attractive pharmacological target [1,3]. Selective irreversible inhibitors of KAT-II, such as PF-04859989 and BFF-122, have been previously developed. PF-04859989 can reduce KYNA levels by 50% in the prefrontal cortex of rats and decrease the firing activity of midbrain dopamine neurons [1,4]. The potential of these inhibitors to treat certain CNS disorders is truly exciting. However, because BFF-122 and PF-04859989 also bind to the enzyme cofactor pyridoxal-5-phosphate (PLP), leading to potentially significant adverse effects, the need for new inhibitors is underscored, preferably competitive inhibitors that do not have these drawbacks [5,6]. Other selective and reversible inhibitors of KAT-II, such as S-ESBA and NS-1502, demonstrated high IC50 values (1000 μM; NS-1502, 315 μM) [6,7]. Supplementing this is the recent inhibitor BFF-816, which induces a decrease in KYNA in the brain after systemic kynurenine injections, but its mechanisms of action on the KAT isoforms are still unclear [6].

Many enzyme inhibitors are derived from natural products, such as flavonoids, widely known for their health benefits. Flavonoids belong to the polyphenol class of phytonutrients. The polyphenol structure of flavonoids accounts for their diverse pharmacological activities, which have shown potential as sources of new antimicrobials, antiviral, antioxidant, and anti-inflammatory drugs [8,9]. Our research takes a unique approach by aiming to identify natural compounds as potential inhibitors of the KAT-II enzyme, which is a less explored avenue in the field [5,10]. We have now discovered new inhibitors of KAT-II through virtual screening of a small library of natural compounds. The molecules were subjected to deep analysis of their ADME proprieties and their stability in the active site of KAT-II using molecular dynamics analysis. The lead compounds were evaluated for their safety profile and inhibitory activity in vitro, demonstrating their potential to impact future drug development.

## 2. Results and Discussion

In recent years, theoretical chemistry and pharmaceutics have seen a growing trend towards using computational approaches in drug design [11]. The aim is to efficiently and cost-effectively identify compounds likely to exhibit physiological activity against specific biologically active macromolecules.

Our research utilized a computational approach to identify two flavonoids as new and safe KAT-II inhibitors from natural compounds. We then conducted in vitro assays to test these molecules’ inhibitory potential and cytotoxicity profile.

### 2.1. Molecular Docking, MMGBSA, and ADMET Analysis

Molecular docking, binding free energy calculation, and drug-likeness analysis are computational methods used to screen large libraries of bioactive compounds and identify potential drug candidates.

Before conducting the virtual screening, we validated the docking procedure using XP docking of the co-crystal ligand through the Glide module. This was done to evaluate the similarity between the lowest energy state predicted by Glide and the observed binding mode of the co-crystal structure (see Figure S1). The root mean square deviation (RMSD) value, a crucial validation metric, between the co-crystal ligand and the re-docked native ligand position was 1.473 Å. An RMSD value of less than 2.0 Å indicates the reliability of the docking procedure [12].

After applying Veber and Lipinski’s rules [13,14] to filter the library compounds, 231 molecules were further screened by XP mode. Then, the final output was filtered for a cutoff value of −7.00 kcal/mol to screen and identify better lead molecules [15,16].

The initial XP GScore output ranged from −1.81 to −8.66 kcal/mol. Only five ligands showed a docking score >−7 kcal/mol, while the remaining molecules had scores between −1.81 and −6.82 kcal/mol, and hence, these were not considered for further analysis.

The top five hits, herbacetin, (-)-Epicatechin, melilotoside, sakakin, and eriodictyol, displayed favorable interactions and binding affinity scores (Table 1).

**Figure 1:**
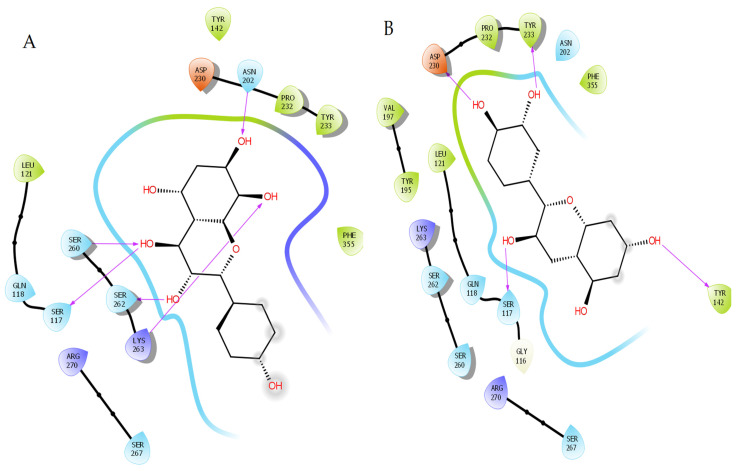

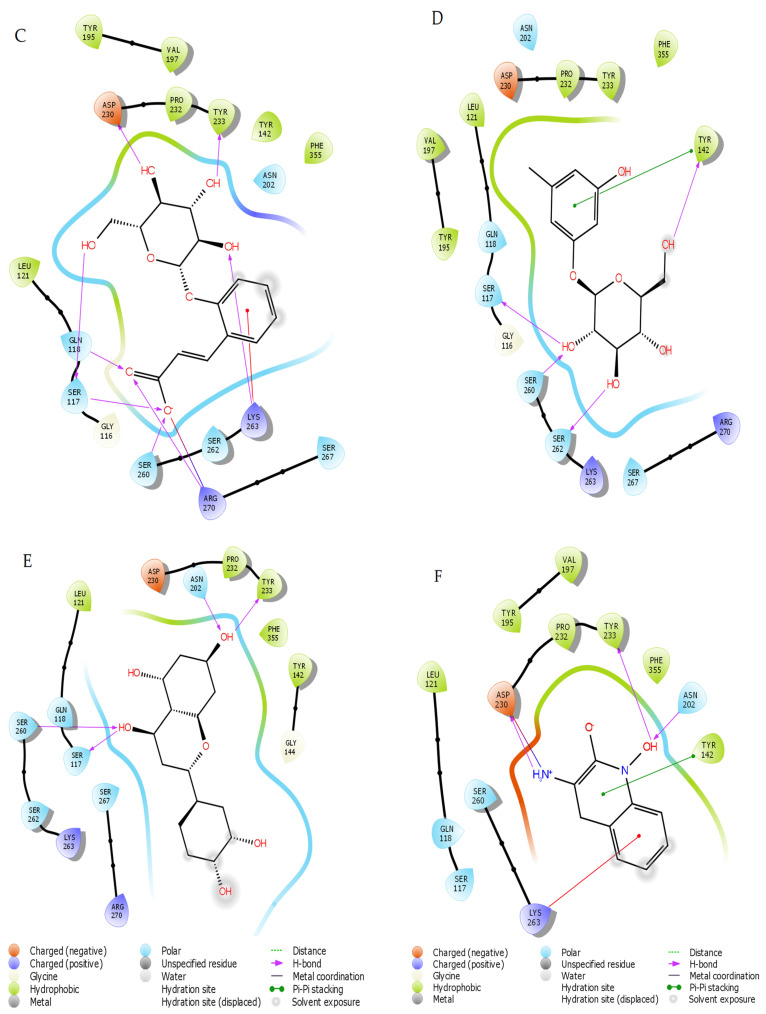
2D interaction of the lead molecules and the standard inhibitor with KAT-II binding site residues; (**A**) herbacetin, (**B**) (-)-Epicatechin, (**C**) melilotoside, (**D**) sakakin, (**E**) eriodictyol, (**F**) PF-04859989.

The structures of ligand-binding pockets, as well as the residues involved in hydrogen-bond formation and hydrophobic interactions, are illustrated in Figure 1 and Table 1 to give more insights into the interactions between KAT-II and the selected compounds.

Herbacetin interacted with binding site residues Asn202, Ser117, Ser260, Ser262, and Lys263 through hydrogen bonds (Figure 1A). The phytocompounds (-)-Epicatechin, melilotoside, sakakin, and eriodictyol also showed significant binding affinities against KAT-II, with docking score values of −8.16 kcal/mol, −7.91 kcal/mol, −7.84 kcal/mol, and −7.636 kcal/mol, respectively (Table 1). (-)-Epicatechin formed hydrogen bonds with the residues Tyr233, Tyr142, Asp230, and Ser117 (Figure 1B). Melilotoside interacted with residues Tyr233, Lys263, Asp230, Ser117, Ser260, and Arg270 through hydrogen bonding (Figure 1C), while sakakin interacted with Tyr142, Ser117, Ser260, and Ser162 (Figure 1D). Eriodictyol, the fifth-best molecule, showed hydrogen bonding with Tyr233, Asn202, Ser260, and Ser117 (Figure 1E). Additionally, other types of interactions, such as hydrophobic interactions, were observed with the target (Figure 1).

Our docking results suggest that the high inhibitory activities of herbacetin and (-)-Epicatechin reflect more significant hydrogen bond interactions with KAT-II active site residues. The previous compounds formed almost the same hydrogen interactions as the recent inhibitor, 17-sulfate of estradiol disulfate, where they can similarly exploit interactions with the conserved residues such as Asn202 and Lys263 [6,7]. According to previous study, the hits with critical residues at the binding site of KAT-II, such as Asn202 and Tyr233, generally interact with the substrate and the cofactor PLP [7,17,18]. Additionally, the residue Lys263 plays a crucial role as a catalytically essential side chain due to its interaction with PLP, which is important to the observed potency of the KAT-II inhibitors [5,6].

On the other hand, the residues required for stabilization of ligand binding by hydrophobic interactions were conserved among herbacetin and compounds (Table 1). In particular, Tyr142 is considered one of the most critical residues for ligand binding.

The compounds with high Glide scores underwent MM-GBSA analysis to calculate binding energies and identify the most promising inhibitors. The five compounds with the highest scoring hits during the molecular docking had binding free energies of −50.30, −51.35, −34.34, −49.51, and −51.33 kcal/mol, respectively. The binding free energy from MMGBSA calculation for PF-04859989 as a reference inhibitor was −38.41 kcal/mol.

### 2.2. Drug-Likeness Predictions and ADMET Analysis

The top five molecules were selected based on docking scores, ligand–receptor interactions, and MMGBSA binding affinity compared with the standard drug PF-04859989.

The overall human oral absorption percentage for the studied molecules and the reference inhibitor ranged from 36.716% to 61.197%. Additionally, these molecules exhibited good octanol–water partition coefficient (QP log Po/w) values, ranging from −0.814 to 0.416 (Table 2).

The blood/brain coefficients (Q log B/B) fell within an acceptable range, between −2.318 and −0.325. The Caco-2 permeability factor (in nm s^−1^) ranged from 5.200 to 98.591, indicating that the studied molecules had good cell membrane penetration (Table 2).

The pharmacokinetic parameters for all compounds, except for melilotoside, fell within the acceptable range specified for human use, as shown in Table 2. Despite the poor Caco-2 permeability value of melilotoside, the compounds are expected to meet Lipinski’s criteria for drug-likeness, with the majority falling within the rule of five. This suggests a high potential for therapeutic development.

The acute toxicity properties of the top compounds were further investigated using the ProToxII online tool. The toxicity parameters were evaluated, including LD50 value, toxicity class, hepatotoxicity, neurotoxicity, immunotoxicity, and mutagenicity (Table 3).

The toxicity classification (class 1 (toxic) to class 6 (non-toxic)) revealed that herbacetin and (-)-Epicatechin demonstrated a safe toxicity profile since they belong to classes 5 and 6. Other compounds, including the reference inhibitor, PF-04859989, lay under toxicity class 4, displaying their toxic nature [19].

Except for herbacetin, which exhibited weak (likely insignificant) mutagenic activity compared to the reference inhibitor, none of the studied compounds demonstrated hepatotoxic, carcinogenic, cytotoxic, immunotoxic, or mutagenic effects, providing further evidence of their safety in potential uses.

### 2.3. Induced Fit Docking Analysis

In this study, we conducted induced fit docking (IFD) on the top two molecules, the main compounds, using PF-04859989 as a reference inhibitor. The results revealed that herbacetin demonstrated the best IFD score of −849.344 kcal/mol, followed by (-)-Epicatechin with an IFD score of −844.853 kcal/mol. These scores were higher than that of PF-04859989, which had an IFD score of −843.776 kcal/mol.

**Figure 2:**
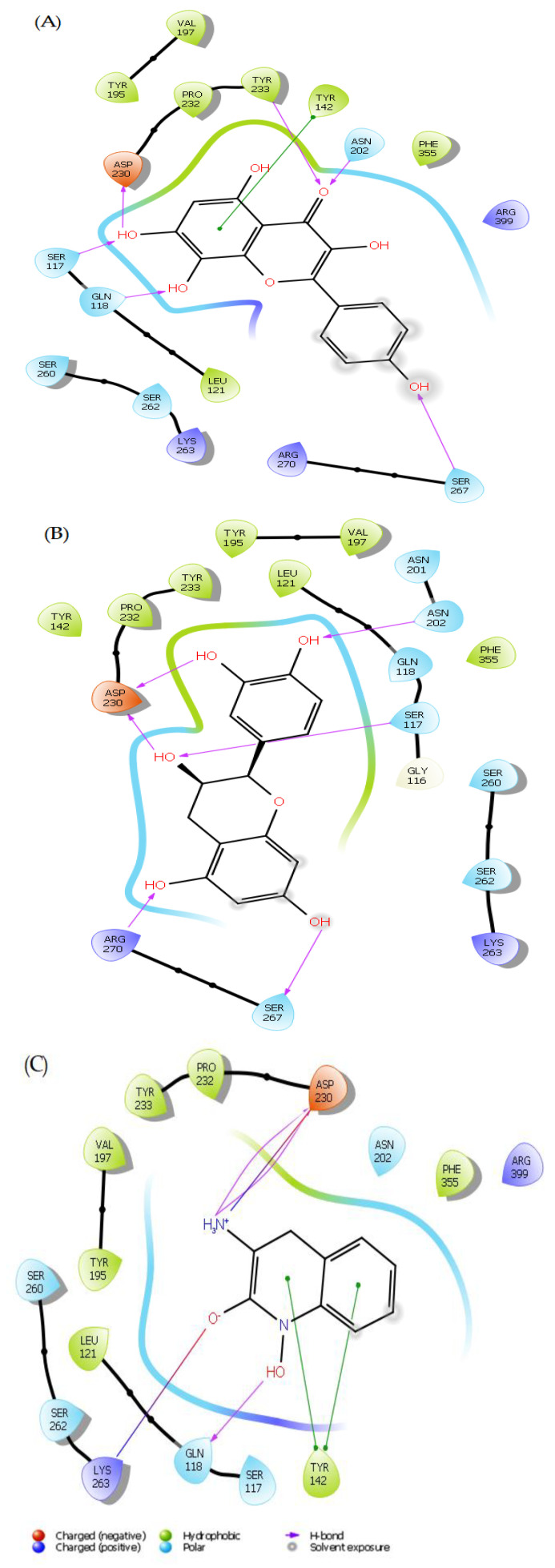
2D interaction diagram of induced fit docking of: (**A**) herbacetin; (**B**) (-)-Epicatechin; (**C**) PF-04859989.

The conformations generated from the IFD showed minimal differences compared to the docked poses achieved through rigid receptor docking. However, new interactions were created or eliminated based on those observed during extra precision (XP) docking. Some ligand interactions remained consistent after IFD docking studies. For the herbacetin compound, new hydrogen bonds were formed with Ser267 and Asp230, but the hydrogen bonds with Ser260 and Lys263 were lost after IFD. Hydrophobic interactions and other hydrogen bonds remained the same as in XP docking (Figure 2A). (-)-Epicatechin formed new hydrogen bonds with Asn202, Ser267, and Arg270, but lost two hydrogen bonds with Tyr142 and Tyr233 (Figure 2B). The standard inhibitor PF-04859989 showed almost the same interaction patterns as XP docking, with a new hydrogen bond with Gln118 and a positively charged amino acid interaction with Arg399 (Figure 2C). Based on these findings, the two lead compounds have a more potent inhibitory effect than PF-04859989, indicating a better interaction with the KAT-II binding site.

### 2.4. Molecular Dynamics Analysis

**Figure 3:**
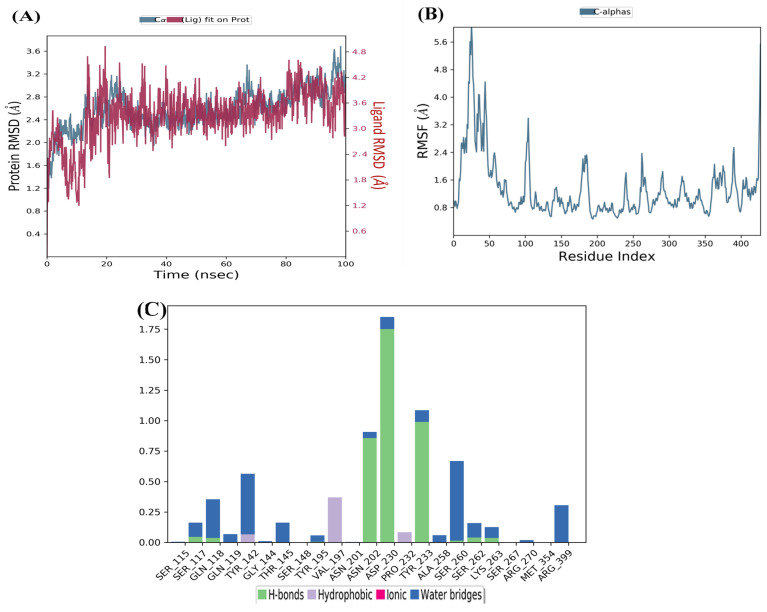
MD simulation for herbacetin–KAT-II complex: (**A**) RMSD plot of herbacetin–KAT-II complex, (**B**) RMSF of herbacetin–KAT-II complex, (**C**) histogram of herbacetin–KAT-II complex.

After analyzing the RMSD graph, we can confidently predict that the herbacetin–KAT-II complex will show minimal RMSD fluctuations throughout the simulation. This stability is evident as the complex remained stable after 20 ns until the end of the simulation, with a maximum value of 2 Å (Figure 3A), which falls within the acceptable range of fluctuations (1–3 Å). This insight into the stable behavior of the herbacetin–KAT-II complex significantly contributes to our understanding of protein–ligand interactions.

**Figure 4:**
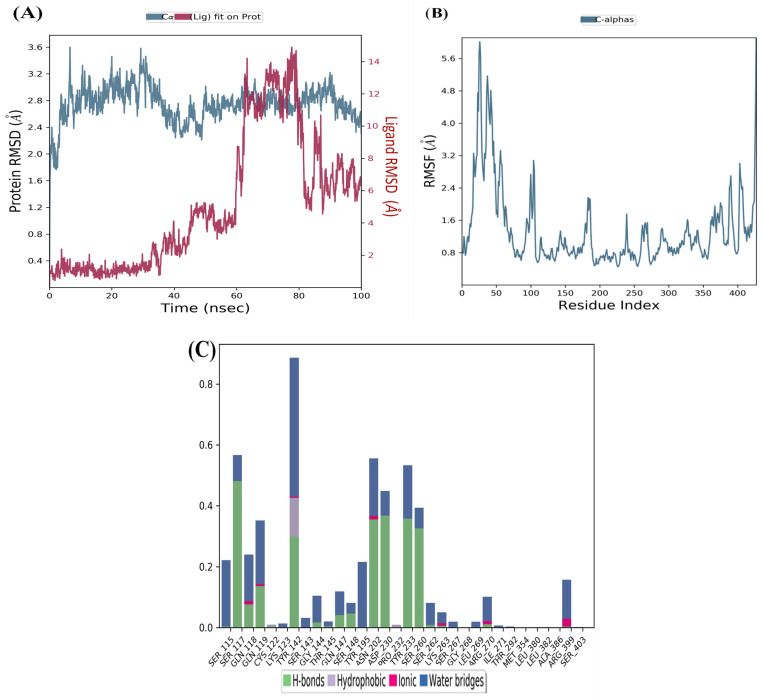
MD simulation for (-)-Epicatechin–KAT-II complex: (**A**) RMSD plot of (-)-Epicatechin–KAT-II complex, (**B**) RMSF of (-)-Epicatechin–KAT-II complex, (**C**) histogram of (-)-Epicatechin–KAT-II complex.

The (-)-Epicatechin–KAT-II complex shows lower fluctuations in its RMSD values, indicating stable conformation behavior during the simulation. Although there is an initial RMSD value of 4 Å and a fluctuation between 0 and 60 ns, the complex stabilizes after 85 ns (see Figure 4A), with both the receptor and ligand RMSD stabilizing.

The RMSF analyses were conducted to assess the flexibility of each residue using the docked complex structures. As depicted in Figure 3B and Figure 4B, the N and C terminals exhibit more significant fluctuations than other parts of the protein. Peaks indicate the highest protein fluctuations and lower fluctuations suggest greater stability of the complexes during simulation. Low fluctuations indicate the system is in equilibrium [12,20].

RMSF was calculated for KAT-II and two potential drug candidates. As depicted in Figure 3B and Figure 4B, the RMSF did not fluctuate significantly over the 100 ns simulation period, and the average RMSF values remained constant for all complexes.

The MD investigation revealed that the lead compound herbacetin displayed hydrogen bonding with residues Tyr233, Asn202, and Asp230 (Figure 3C). Hydrophobic contacts between the ligand and Tyr142, Ser260, and Arg 399 were also observed. The simulation also showed binding interactions between (-)-Epicatechin and the active site residues of KAT-II, including hydrogen bonding with Ser117, Tyr142, Tyr233, Asn202, and Asp230, as well as hydrophobic interactions with Ser115 and Tyr195 (Figure 4C).

### 2.5. In Vitro Inhibition and Kinetics Study

**Figure 5:**
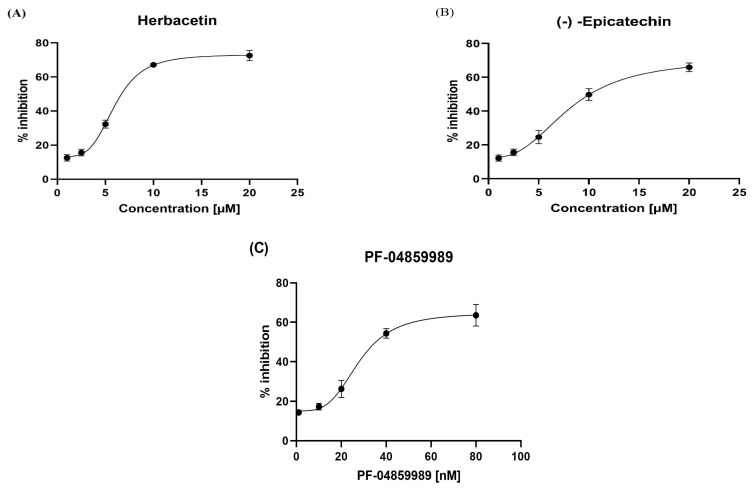
Inhibitory activity of (**A**) herbacetin, (**B**) (-)-Epicatechin, and (**C**) PF-04859989 compounds in a dose-dependent manner. All experiments were performed in triplicate and plotted using GraphPad Prism v8.4.0.

Herbacetin and (-)-Epicatechin displayed a good inhibitory potential of KAT-II at the concentrations used, with IC50 values of 5.98 ± 0.18 µM and 8.76 ± 0.76 µM, respectively. (Figure 5A,B). However, the standard, PF-04859989, was the most effective inhibitor (IC50 value 27.91 ± 1.81 nM; *p* < 0.05). Additionally, we compared the inhibitory characteristics of these compounds with those of PF-04859989, which bind to PLP irreversibly (Table S1). From this analysis, it is apparent that herbacetin and (-)-Epicatechin exhibit reversible inhibition. This behavior was confirmed by varying the PLP concentration in the reaction mixture, resulting in decreased inhibition by the studied compounds as the PLP concentration increased, indicating competition [6,12].

**Figure 6:**
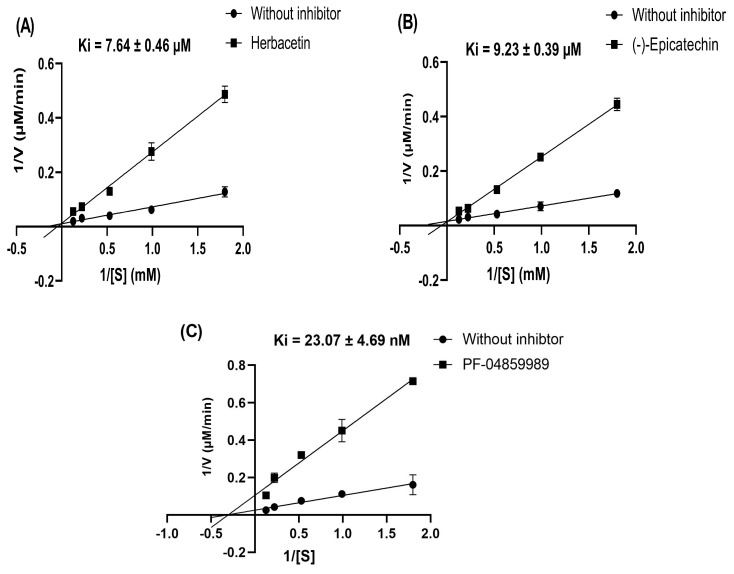
Lineweaver–Burk plots of inhibitory kinetics of herbacetin and (-)-Epicatechin towards KAT-II. Kinetics parameters of (**A**) herbacetin, (**B**) (-)-Epicatechin, and (**C**) PF-04859989 were evaluated using Lineweaver–Burk analysis. All experiments were performed in triplicate, and data are presented as mean ± SD.

**Figure 7:**
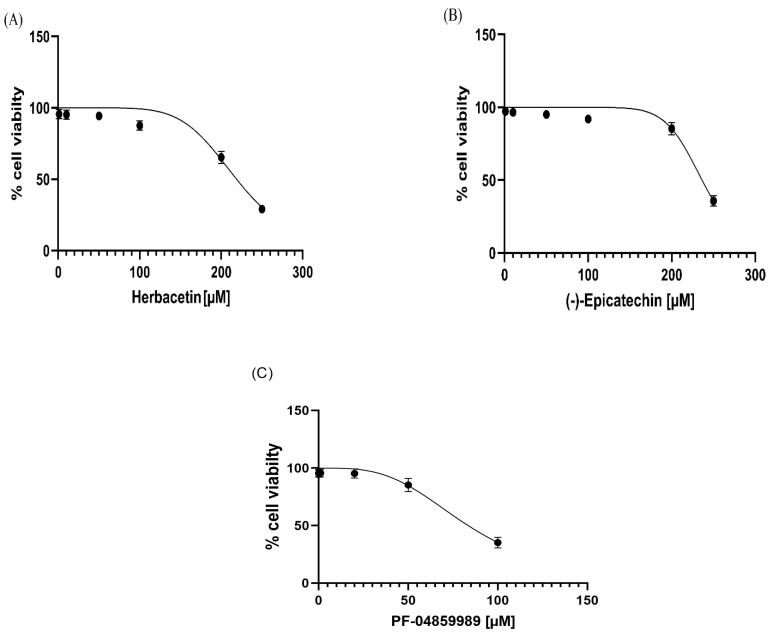
Cell viability (%) of HepG2 cells, measured by the MTT assay, after 72 h exposure to (**A**) herbacetin, (**B**) (-)-Epicatechin, and (**C**) PF-04859989.

The inhibitory mechanisms showed that herbacetin and (-)-Epicatechin are competitive inhibitors since the value of the Michaelis constant (Km) changed with substrate concentrations and the maximum velocity (Vmax) did not vary in the presence of increasing concentrations of the inhibitors (Figure 6A,B). However, in the case of the reference inhibitor, PF-04859989, Vmax decreased while Km remained unaffected, indicating a non-competitive type of inhibition (Figure 7C).

The obtained IC50 clearly demonstrated that herbacetin and (-)-Epicatechin strongly inhibited KAT-II when compared to the inhibition profiles of the synthetic reversible inhibitor NS-1502 (IC50: 315 μM) and the irreversible inhibitor BFF-122 (IC50: 15–20 μM), which were designed using kynurenine as a scaffold [6,21,22]. The two selected compounds were found to be reversible. As the concentration of PLP increased, the inhibitory effect of these compounds decreased, indicating that they competed with PLP. This contrasts with the irreversible inhibitor PF-04859989, which was used as a positive control and displayed similar inhibition as PLP concentration changed. Likewise, our results indicate that our reversible inhibitors inhibit KAT-II in a similar way to the several natural inhibitors recently identified, including glycyrrhizic acid, glycyrrhetinic acid, and carbenoxolone, which reversibly bind to PLP and exhibit an IC50 of 4.51 ± 0.20 µM, 6.96 ± 0.37 µM, and 3.90 ± 0.37 µM, respectively [23].

Therefore, we identified two novel natural inhibitors that surpass existing KAT-II inhibitors, particularly regarding their favorable pharmacokinetic profile, which was not considered in previous studies.

### 2.6. Cytotoxicity Evaluation

We conducted a test to measure the impact of different concentrations (1–250 µM) of the top two compounds on cell viability using an MTT (3-(4,5-dimethylthiazol-2-yl)-2,5-diphenyltetrazolium bromide, a tetrazole) assay with human hepatocellular carcinoma cells (HepG2). Herbacetin and (-)-Epicatechin showed minimal impact on inhibiting cell growth, with IC50 values of 218.90 µM ± 4.53 and 236.40 ± 2.53, respectively (Figure 7A,B). The standard inhibitor, PF-04859989, seems more cytotoxic when compared to the previous compounds and exhibited an IC50 value of 83.49 ± 2.86 µM (*p* < 0.05) (Figure 7C). Hence, our results indicated that the compounds are not cytotoxic against the HepG2 cell line, particularly at higher concentrations (>200 µM).

Our results from computer simulations and in vitro experiments suggest interactions between the compounds studied and the target protein may exist. However, further extensive experimental validation is necessary to confirm their pharmacological relevance. These computational findings are hypotheses and not definitive evidence of therapeutic effectiveness. To progress from computer simulations to developing clinically useful KAT II inhibitors, conducting in vivo studies to assess safety, pharmacokinetics, and pharmacodynamics, is essential.

## 3. Materials and Methods

### 3.1. Chemicals and Reagents

The recombinant KAT-II protein and buffers were bought from R&D Systems, Bio-Techne (Abingdon Science Park, Abingdon, UK). HepG2 cell line, solvents, and other chemicals, including herbacetin, (-)-Epicatechin, and PF-04859989, were obtained from Sigma Aldrich, LLC (Germany) (Sigma-Aldrich Chemie, Eschenstr, Taufkirchen, Germany). 

### 3.2. Computational Methods

#### 3.2.1. Protein Preparation and Receptor Grid Generation

The crystal structure of human KAT-II (resolution: 2.89 Å; PDB ID: 4GDY) [24] was downloaded from the RCSB Protein Data Bank (RCSB PDB) [25]. The protein sequence is 439 amino acids long and is associated with a co-crystal ligand (native inhibitor). Before molecular docking, we used the Protein Preparation Wizard in Maestro Schrödinger to optimize the KAT-II structure. The PLP cofactor was removed due to possible competition with the ligands at the active site [6]. Water molecules not involved in these interactions were removed, and hydrogen atoms were added. Partial charges were assigned using the OPLS-3e force field in Maestro. The binding site was defined using the receptor grid generation tool. The enclosing cubic grid was centered at coordinates (*x* = 4.15, *y* = 39.13, *z* = 26.61), and the maximum length for ligands to be docked was set to 15 Å.

#### 3.2.2. Ligands Preparation

We started with 480 natural compounds from the Sigma-Aldrich natural products portfolio (https://www.sigmaaldrich.com/DZ/en/products/chemistry-and-biochemicals/biochemicals/natural-products) (accessed on 23 April 2023) to create a small library. The 3D structures of these molecules and the reference inhibitor, PF-04859989, were obtained in SDF (structure data format) from the PubChem database [26]. We then carefully filtered these molecules according to the Lipinski and Veber rules [13,14] using the Ligand Filtering tool in the Schrodinger suite. This process resulted in 231 compounds that met all the selection criteria and were chosen for virtual screening. The Ligprep module in Maestro Schrödinger was used to prepare the ligands. The ionization states at pH 7.0 ± 2.0 and tautomeric forms of each molecule were generated.

#### 3.2.3. Molecular Docking Based Virtual Screening

In this study, we conducted a virtual screening using a library of natural products obtained from the Sigma-Aldrich portfolio to evaluate their potential as inhibitors against one of the main enzymes of the Kynurenine pathway.

The chosen molecules were screened against KAT-II using the Glide program of Schrödinger, LLC (New York, NY, USA, v2021.2). All phyto-compounds were docked using the Glide XP (extra precision) mode [27]. The top five molecules, retaining only the best scoring states, were selected based on docking score and molecular interactions with the target protein for further analysis. Finally, the Pose Viewer (in Maestro v 12.8.117) was used to analyze the interactions of various ligand and protein complexes.

#### 3.2.4. MMGBSA Calculation

The Prime/MMGBSA calculation was applied for rescoring the docked ligand and provided the relative binding free energy (ΔG bind) for each docked ligand pose. These poses were then subjected to energy minimization of the protein–ligand complexes. The binding free energy (ΔG bind) of the top five molecules (hits) was estimated using the following equation:

ΔG (binding affinity) = ΔG (solvation energy) + ΔE (minimized energy) + ΔG (surface area energies)

Here, ΔG (solvation energy) represents the difference between the solvation energy of the GBSA of the inhibitor-KAT-II complex and the sum of the solvation energies for unligated KAT-II and the respective inhibitor. ΔE (minimized energy) is the difference in energy between the complex structure and the sum of the energies of KAT-II with and without the inhibitor, and (ΔG Surface area) is the difference in the energy of the surface area for the inhibitor-KAT-II complex and the sum of the surface area energies for the inhibitor and unbound protein.

#### 3.2.5. Drug Likeness Predictions and ADMET Analysis

The QuikProp module of the Schrodinger suite (Schrodinger, LLC, New York, NY, USA, v2021.2) was used to analyze ADME and drug-likeness properties of selected molecules along with the standard inhibitor by checking for any violation of Lipinski’s rule of five (ROF) and several pharmacological parameters including human Caco2 cellular permeability, oral absorption, and predicted octanol/water partition coefficient. The toxicity parameters were estimated via the ProTox-II web tool (https://tox-new.charite.de/protox_II/) [19].

#### 3.2.6. Induced Fit Docking (IFD)

The IFD method was used to simulate the flexibility of the receptor and the ligand using the induced fit docking function in Maestro v12.8. (Schrodinger, LLC). Removing false negative bonds almost produces an exact binding state comparable to biological ligand–receptor binding.

Based on the XP-docking score, ligand interactions, and binding free energy, the two top molecules were subjected to the IFD method to improve docking accuracy and allow conformational flexibility of ligands and proteins, which is restricted in docking studies.

A standard protocol in the first step of IFD was employed to create the box in the centroid of the ligand at the 0X1 native ligand and 4GDY receptor binding site. Then, for each ligand, up to 20 docked poses were generated for refinement by Prime within 5, with receptor and ligand van der Waals radii of 0.7 and 0.5, respectively. After prime side-chain prediction and minimization, the final docked poses for each ligand were returned to Glide SP for redocking.

#### 3.2.7. Molecular Dynamics (MD)

We conducted a molecular dynamics (MD) simulation over a 100 ns time scale to assess the stability of the top two docked complexes, herbacetin and (-)-Epicatechin in the active site of KAT-II. We used Glide XP docking to investigate the expected binding mode and types of interactions. To evaluate the simulations, we examined the root mean square deviation (RMSD), root mean square fluctuation (RMSF), and protein–ligand contacts. The RMSD helps us understand the conformational stability of a structure during simulation by measuring the average change in atom displacement compared to a reference.

Protein Wizard was used to prepare the protein–ligand complex structure of KAT-II and the two top candidate molecules for MD simulation. Desmond-2018 version was used to conduct 100 ns and generate 1000 frames. The complex protein–ligand interaction was placed in an SPC (single point charge) water box of size 10 Å × 10 Å × 10 Å, under orthorhombic periodic boundary conditions. The System Builder tool was used to prepare the buffer, which was then neutralized by adding 0.15 M NaCl ions. In the next step, the energy minimization was conducted by 2000 steps using the steepest descent method with a cutoff of 9 Å for van der Waals interactions. Also, the NPT ensemble was applied to maintain the system’s temperature (K) and pressure (bar) at 300 K and 1.01325 bar, respectively, throughout the MD experiment. The force field OPLS3e was used to do all the runs [12].

### 3.3. In Vitro Inhibition and Kinetics Study

The KAT-II inhibition test was conducted based on the method reported by Lu et al. [28], with a slight modification. We performed the assay on the two most promising molecules using a microplate fluorescent test for kynurenine aminotransferase. The reaction mixture (20 μL) contained 20 nM recombinant human KAT-II, 1 mM L-KYN, 0.3 mM L-α-aminoadipic acid (AAD), 50 µM α-ketoglutaric acid, 3 mM NAD+, and 88 µg/mL glutamic dehydrogenase.

The inhibitory activity of the two molecules, herbacetin and (-)-Epicatechin, was tested at different concentrations after being diluted in DMSO. These molecules were pre-incubated with the enzyme for 30 min at room temperature in a 0.1 M phosphate buffer at pH 7.5 containing 5 µM PLP. Subsequently, kinetic data were collected over 30 min. The percentage of inhibition was determined for each inhibitor concentration, and the IC50 value was calculated through non-linear regression (four parameters). The assay was validated using the reference inhibitor PF-04859989, and its IC50 (IC50 = 27.91 ± 1.83 nM) was consistent with the value reported by Maryška et al. [10].

The kinetics study of the herbacetin and (-)-Epicatechin was quantitatively analyzed using Lineweaver–Burk plots (double reciprocal plots) to investigate the type of inhibition. We were using the same procedure as the enzyme inhibition assay. The concentration used of the substrate (L-Kyn) range was 1.00, 0.8, 0.60, 0.40, and 0.20 mM, and herbacetin and (-)-Epicatechin at 1.00, 10.00, and 20.00 μM, respectively. The competitive inhibition constant (Ki) for herbacetin and (-)-Epicatechin was calculated using GraphPad Prism (v8.4.0).

### 3.4. Cytotoxicity Assay

The MTT assay assessed cell cytotoxicity, following the method described by [29] with slight modifications. The HepG2 cell line was obtained from ECACC (European Collection of Authenticated Cell Cultures, Sigma-Aldrich, Germany). Initially, the cells were cultured in DMEM medium containing 10% fetal bovine serum (FBS), 2 mM L-glutamine, 1 mM sodium pyruvate, 100 U/mL penicillin, 100 μg/mL streptomycin, and 250 μg/mL fungizone, in a humidified incubator at standard conditions of temperature (37 °C) and CO_2_ (5%). Cells were rinsed with 2.5 mL PBS and treated with 1.5 mL 0.25% trypsin-EDTA for 5 min at 37 °C. Cell pellets were collected by spinning at 1500 rpm for 6 min at 4 °C, then resuspended in fresh culture media with the supernatant replaced; the culture medium was refreshed with fresh media every other day. Three wells were used in the 96-well plates for each molecule, with repetitions carried out three times.

Cell viability was determined using the MTT (3-(4,5-dimethylthiazol-2-yl)-2,5-diphenyltetrazolium bromide) tetrazolium reduction assay. The cells were dispensed at 8000 cells/well in 0.1 mL medium. Cells were treated with herbacetin and (-)-Epicatechin (dissolved in DMSO) with variable concentrations from 1–250 µM and incubated at 37 °C for 72 h. Then, 10 microliters of MTT solution at a concentration of 5 mg/mL were introduced into every well, followed by incubating the plates at 37 °C for 4 h. The reduced MTT’s absorbance was recorded at 570 nm using a VERSA max microplate reader.

Cell viability data of the studied molecules were plotted against concentrations of derivatives, and the IC_50_ values were determined graphically using the curve-fitting algorithm.

### 3.5. Statistical Analysis

Statistical analysis was performed using GraphPad Prism 8.4.0. Experimental procedures were carried out in triplicate (n = 3), and the results were presented as the mean ± standard deviation (SD).

## 4. Conclusions

In this study, we rely on the effectiveness of computational methods and the in vitro kinetics study to identify new potential and safe inhibitors for KAT-II. The molecular docking identified two potential drug candidates, herbacetin and (-)-Epicatechin. Both compounds showed good binding affinity with KAT-II. Molecular dynamics results were also in agreement with docking results and indicated suitable stability and proper interaction during the time for both ligands. The findings also revealed acceptable ADME properties and a safe molecule toxicity profile. Furthermore, the in vitro assays indicated that the selected molecules act as competitive and reversible inhibitors exhibiting a potent inhibitory activity against KAT-II and a non-toxic effect on cell viability. We consider these compounds excellent candidates for the effective treatment of schizophrenia, but further preclinical trials are needed.

## Figures and Tables

**Table 1:** Energy-based interactions detail of the top five hits.

Compounds	Docking Score (kcal mol^−1^)	Glide Emodel (kcal mol^−1^)	MMGBSA Δ*G* Bind (kcal mol^−1^)	Number of H-Bond Formation	Amino Acid Interactions and H-Bond Distance (Å)
Herbacetin	−8.66	−44.95	−50.30	5	Asn202 and Ser117 (3.0 Å and 2.01 Å) Ser260 and Ser262 (1.98 Å and 2.01 Å) Lys263 (2.54 Å)
(-)-Epicatechin	−8.16	−39.32	−51.35	4	Tyr233 and Tyr142 (1.82 Å and 2.0 Å) Asp230 and Ser117 (1.93 Å and 2.02 Å)
Melilotoside	−7.91	−51.41	−34.34	6	Tyr233, Lys263 (1.89 Å and 2.74 Å) Asp230 and Ser117 (1.77 Å and 2.21 Å) Ser260 and Arg270 (1.99 Å and 1.73 Å)
Sakakin	−7.84	−44.76	−49.51	4	Tyr142 and Ser117 (2.12 Å and 1.96 Å) Ser260 and Ser262 (2.07 Å and 1.79 Å)
Eriodictyol	−7.63	−42.53	−51.33	4	Tyr233 and Asn202 (2.02 Å and 2.09 Å) Ser260 and Ser117 (2.33 Å and 2.02 Å)
PF-04859989 (reference inhibitor)	−7.12	−28.88	−38.41	3	Tyr233 and Asn202 (2.04 Å and 2.73 Å) Asp230 (1.81 Å)

**Table 2:** ADME properties of top five molecules.

Parameters	Herbacetin	(-)-Epicatechin	Melilotoside	Sakakin	Eriodictyol	PF-04859989
HB donor	4.000	5.000	5.000	5.000	3.000	3.000
HB acceptor	5.250	5.450	11.250	10.000	4.750	5.200
% of human oral absorption	53.100	61.197	36.716	56.651	62.522	59.55
QP log P_0_/w	0.416	0.454	−0.520	−0.814	0.875	−0.525
QP log S	−2.846	−2.518	−1.886	−1.751	−3.930	−0.233
QPPCaco	27.141	58.241	5.200	84.334	50.276	98.591
QP log B/B	−2.318	−1.815	−2.682	−1.866	−1.797	−0.325
Rule of five	0	0	0	0	0	0

HB donor: hydrogen bond donor, HB acceptor: hydrogen bond acceptor, % human oral absorption: >80% is high and <25% is poor, QP log Po/w: −2.0–6.5, QP log S: −6.5–0.5, QPPCaco: <25 poor and >500 excellent, QP log B/B: predicted brain/blood partition coefficient; −3.0–1.2, Rule of five: no. of violations of Lipinski’s rule of five (0 is good and 4 is bad).

**Table 3:** Toxicity assessment of the candidate compounds.

Parameters		Herbacetin	(-)-Epicatechin	Melilotoside	Sakakin	Eriodictyol	PF-04859989
LD_50_ (mg/kg)		3919	10,000	1500	1380	2000	500
Prediction class		Class 5	Class 6	Class 4	Class 4	Class 4	Class 4
Hepatotoxicity	Prediction	Inactive	Inactive	Inactive	Inactive	Inactive	Inactive
	Probability	0.69	0.72	0.82	0.92	0.67	0.53
Neurotoxicity	Prediction	Inactive	Inactive	Inactive	Inactive	Inactive	Active
	Probability	0.89	0.90	0.88	0.92	0.88	0.57
Immunotoxicity	Prediction	Inactive	Inactive	Active	Inactive	Inactive	Inactive
	Probability	0.92	0.96	0.56	0.96	0.71	0.99
Mutagenicity	Prediction	Active	Inactive	Inactive	Inactive	Inactive	Active
	Probability	0.51	0.55	0.78	0.76	0.59	0.62
Prediction accuracy		70.97%	100%	69.26%	69.26%	69.26%	68.07%

Prediction class: class 4 (harmful if swallowed (300 < LD50 ≤ 2000); class 5: may be harmful (slightly toxic) if swallowed (2000 < LD50 ≤ 5000); class 6: non-toxic (LD50 > 5000) [19].

## Data Availability

The data presented in this study are available on request from the corresponding author.

## Supplementary Materials

The following supporting information can be downloaded at: https://www.mdpi.com/article/10.3390/ph18010076/s1, Figure S1: Validation of docking study; Figure S2: 2D structure of lead compounds and the standard inhibitor; Figure S3: 3D interactions of the lead molecules and the reference inhibitor with KAT-II binding site residues; (A) herbacetin, (B) (-)-Epicatechin, (C) melilotoside, (D) sakakin, (E) eriodictyol, (F) PF-04859989; Table S1: Inhibition type of herbacetin, (-)-Epicatechin, and PF-04859989 on KAT-II.
