# Supplementary material for: Identification of Two Flavonoids as New and Safe Inhibitors of Kynurenine Aminotransferase II via Computational and In Vitro Study
Source: Pharmaceuticals (Basel). 2025 Jan 10;18(1):76. doi: 10.3390/ph18010076 (PMC11768104; doi:10.3390/ph18010076)
Supplement: Supplementary file 1 [file pharmaceuticals-18-00076-s001.zip › pharmaceuticals-3394684-supplementary.pdf]

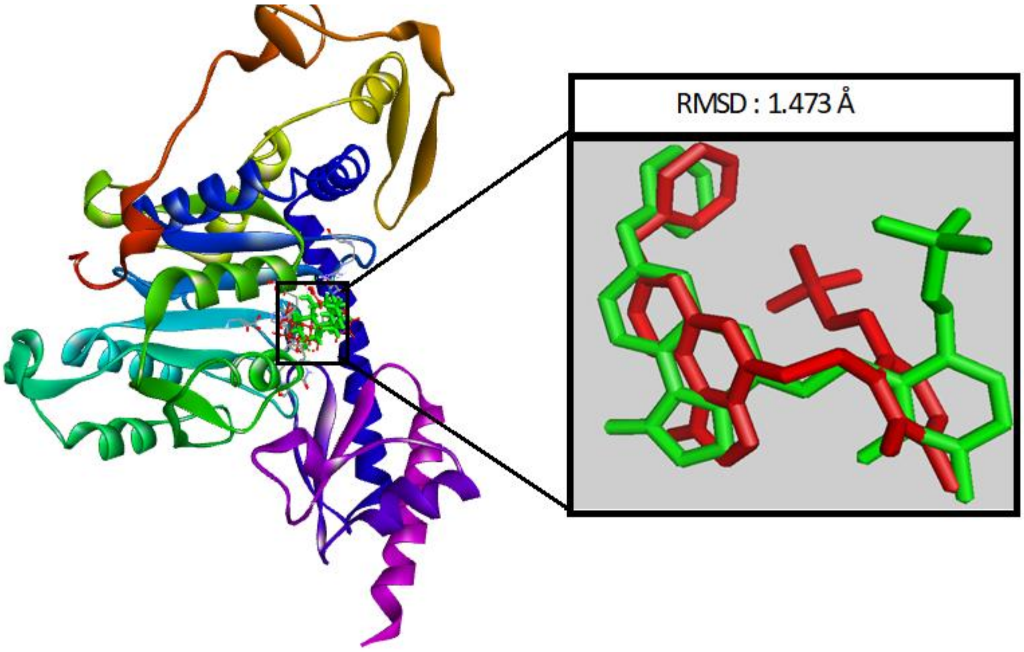

FigureS1: Superimposition of the docked co-crystal ligand (in red color) and the co-crystal ligand (in green color) with RMSD of 1.473Å, (validation of docking study).

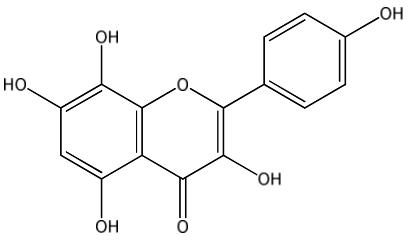

Herbacetin

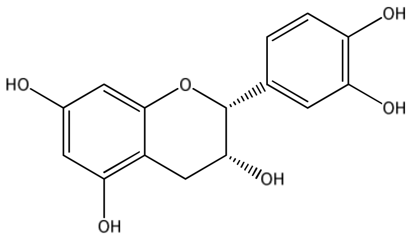

(-)-Epicatechin

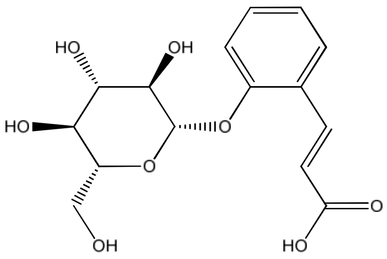

Melilotoside

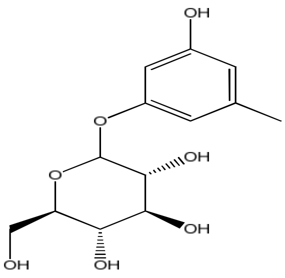

Sakakin

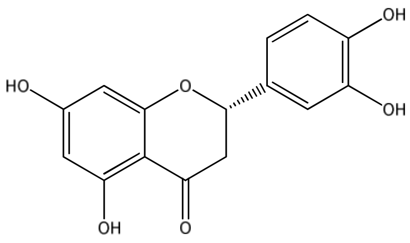

Eriodictyol

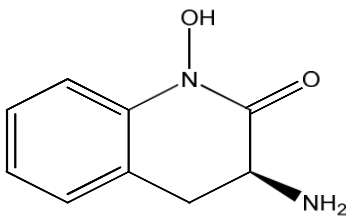

PF-04859989

Figure S2: 2D structure of lead compounds having potential to bind at the protein-binding site and the standard inhibitor (PF-04859989).

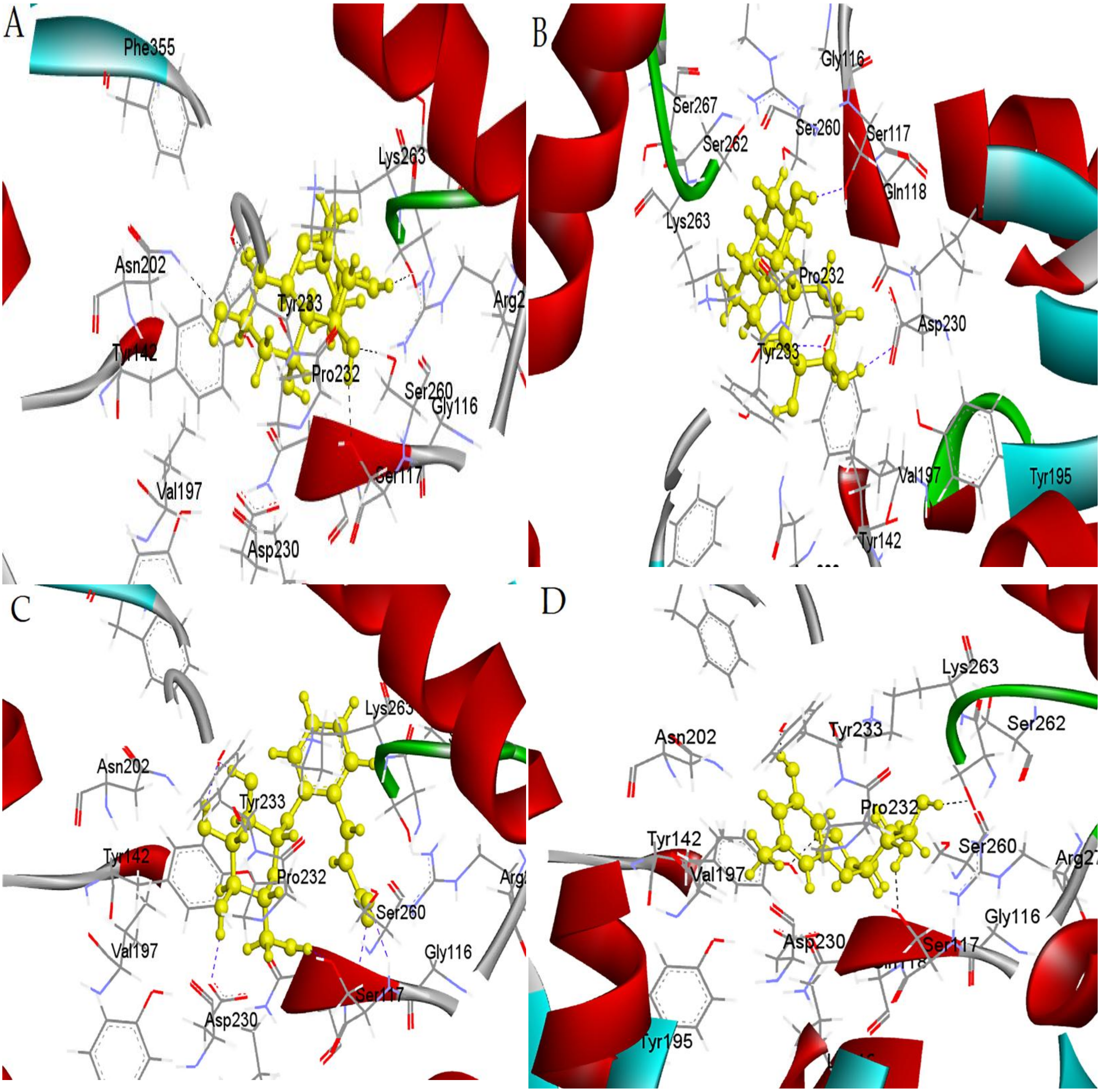

A
Phe355
Lys263
Asn202
Tyr233
Arg2
Tyr142
Pro232
Ser260
Gly116
Ser117
Val197
Asp230
B
Gly116
Ser267
Ser260
Ser117
Ser262
Gln118
Lys263
Pro232
Asp230
Tyr233
Val197
Tyr195
Tyr142
C
Lys263
Asn202
Tyr233
Tyr142
Pro232
Arg
Ser260
Val197
Gly116
Ser117
Asp230
D
Lys263
Asn202
Tyr233
Ser262
Pro232
Tyr142
Ser260
Arg27
Val197
Gly116
Asp230
Ser117
Tyr195

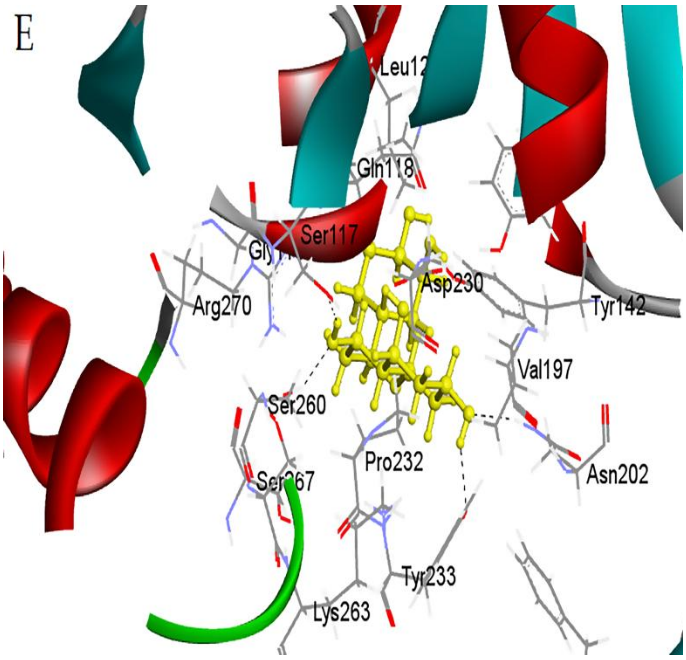

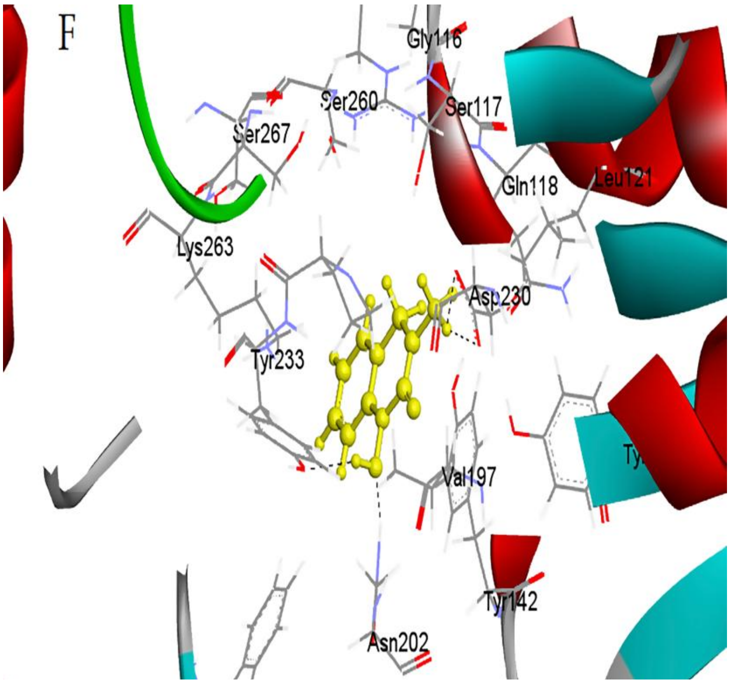

Figure S3: 3D interactions of the lead molecules and the reference inhibitor with KATII binding site residues; (A): Herbacetin, (B): (-)-Epicatechin, (C):Melilotoside, (D): Sakakin, (E): Eriodictyol, (F):PF-04859989.

TableS1: Inhibition type of herbacetin, (-)-EpicatechinandPF-04859989onKAT-II

PLP concentration(μM)	Herbacetin (Inhibition %)	(-)-Epicatechin (Inhibition %)	PF-04859989 (Inhibition %)
5	72.58±2.98	69.25±4.42	63.58±5.47
10	69.71±1.94	63.08±4.92	61.67±1.74
50	59.25±3.10	50.81±0.94	62.91±4.27
100	45.34±3.79	42.65±2.86	64.10±3.38
